# A DNA-Binding Protein Helps Repair Breaks in DNA Double Helix

**DOI:** 10.1371/journal.pbio.0020074

**Published:** 2004-01-20

One of the central problems for much of the 20th century was how to reconcile genetic stability with evolutionary change. Genomic fidelity was thought to arise from an inherent invariability in the DNA structure itself. Biologists now know that DNA constantly undergoes modifications as it unwinds, replicates, condenses, twists, and untwists. This dynamic interplay produces both stability and variation—and occasionally genetic damage. If DNA damage goes unrepaired, it can disrupt chromosomal integrity and may lead to cancer and other diseases. When the DNA double helix breaks, the cell must enlist a number of proteins to repair the broken DNA ends, but much remains to be learned about the molecular mechanisms involved. Tracking a protein that binds to single strands of DNA during replication and recombination in living yeast cells, Xuan Wang and James Haber report that this protein plays a role in at least two key steps in the repair of double-strand breaks in DNA.

When double-strand breaks occur, the cell mounts a search for similar (homologous) sequences that can be used as a template to repair the damaged sequence. If successful, the broken DNA molecule basepairs with the homologous region and forms a complex, ultimately replacing the damaged sequence with a similar sequence. In yeast—which serves as a stand-in for higher eukaryotes, including humans—this “strand invasion” process requires both an exchange protein, called Rad51, and a single-stranded DNA-binding protein, called RPA (replication protein A). Single-stranded binding proteins bind to regions of DNA that are opened up during replication. They also bind to strands when broken ends of DNA are cut by enzymes that leave long single-stranded tails. RPA proteins are thought to facilitate the formation of Rad51 polymers, or filaments, on single-stranded DNA by clearing away structures that block Rad51's path. The growing filament searches for homologous DNA sequences and promotes the invasion of the single strand, preparing it to copy the homologous template by “repair DNA synthesis,” which patches up the lesion.

To investigate how RPA functions in double-strand break repair in a living organism, Wang and Haber created cells with a double-strand break at a specific site and monitored the activity of proteins recruited to repair the damage. With this approach, the researchers could observe these interactions in living yeast to determine what role RPA plays in repairing DNA damage and how it works with the Rad51 protein.

The authors show that as soon as a double-strand break occurs, the RPA protein binds to the exposed strand ends, before the Rad51 protein does. This is not unexpected, because this binding order supports the model that RPA prepares the way for Rad51, perhaps by stabilizing the strand long enough for Rad51 filaments to establish themselves. The surprise was that RPA appears to be necessary even after Rad51 binds to the DNA strand, perhaps by stabilizing the interaction with homologous DNA sequences. That RPA is required for successful repair is supported by evidence that a particular mutated form of RPA can stimulate Rad51 DNA binding normally, but inhibits strand exchange and template copying, thus preventing repair of DNA damage.

Wang and Haber's work highlights the complex repertoire of DNA–protein and protein–protein interactions that manage and manipulate the genome in the service of genomic stability. The study of DNA repair mechanisms in living cells—a daunting task—promises to lend valuable insights into the truly dynamic nature of maintaining genome stability.

**Figure:**
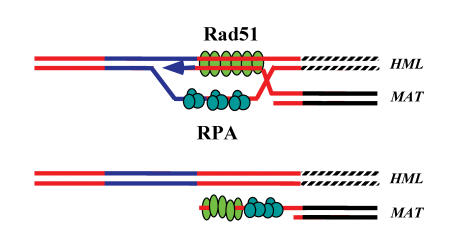
Repair of double-strand breaks involves invasion of the homologous region, displacement, and DNA synthesis to fill in the gap

